# Acute Upper Limb Ischemia as a Presentation of Arterial Thoracic Outlet Syndrome in a Young Patient: A Case Report

**DOI:** 10.7759/cureus.107438

**Published:** 2026-04-21

**Authors:** Arianna Grilli, Auréline Cousinne, Fulvio Massaro, Youri Sokolow

**Affiliations:** 1 General Surgery, Université Libre de Bruxelles, Brussels, BEL; 2 Surgery, Université Libre de Bruxelles, Brussels, BEL; 3 Hematology, Institut Jules Bordet, Brussels, BEL; 4 Thoracic Surgery, Erasmus Hospital, Brussels, BEL

**Keywords:** acute upper limb ischemia, arterial thoracic outlet syndrome, cervical rib, cervical rib resection, subclavian artery thrombosis

## Abstract

Thoracic outlet syndrome (TOS) refers to a group of conditions caused by extrinsic compression of neurovascular structures at the thoracic outlet. Arterial TOS (aTOS) is the rarest form and may present with upper limb ischemic symptoms such as pain, coldness, pallor, and numbness, most often due to bony abnormalities such as a cervical rib.

We report the case of a 19-year-old woman on combined oral contraceptive therapy who presented with acute ischemia of the right upper limb. Duplex ultrasound and computed tomography angiography revealed thrombosis of the right subclavian artery in the setting of a right cervical rib. The patient underwent urgent thrombectomy followed by definitive decompression through supraclavicular resection of the supernumerary rib.

This case illustrates a rare presentation of aTOS in a young patient and highlights the importance of prompt recognition and appropriate surgical management.

## Introduction

Thoracic outlet syndrome (TOS) has a wide definition that includes all conditions characterized by upper limb symptoms caused by a compression of neurovascular structures located in the area just above the first rib. While there are many ways to classify this syndrome, the preferred one is the anatomical: whether the main affected structure is nervous or vascular, this syndrome has been divided into three main branches, called neurogenic TOS (nTOS), venous TOS (vTOS), and arterial TOS (aTOS) [1].

The aTOS is the rarest manifestation of the three, as it appears in less than 1% of all TOS patients [2-4]. Males and females are equally affected by it [3,4].

The etiologies of aTOS can be congenital or acquired. Congenital causes are typically associated with bony abnormalities, mostly cervical ribs (64%) or anomalous first ribs (17%), but also enlarged C7 transverse process (1%) and fibrocartilaginous bands from the anterior scalene muscle (11%) [4]. Whereas acquired causes are clavicular fracture (7%) [4] with malunion or prominent bony callus [5] or muscle hypertrophy in athletes [4].

Concerning its main etiology, cervical ribs and anomalous first ribs, based on a study published in 1939, have been determined to have an annual incidence of 0.76% and 0.74% respectively, with no difference between men and women for the anomalous ribs, whereas for cervical ribs, it has been showed a 70% majority in women [6]. Most of these abnormalities are, in fact, asymptomatic but can represent a risk factor for the development of aTOS [2].

Most cases of aTOS are asymptomatic [4]. In symptomatic cases, it shows with pain, paresthesia, claudication, coldness, color changes, or even digital gangrene, typical of ischemia of the extremities [2]. Clinical signs that may be observed are: the absence of brachial pulse, and sometimes also of the ulnar and radial pulses, a systolic blood pressure difference between the two arms up to 20 mmHg [3], and motor and/or sensitive deficits [4].

The affected artery is the subclavian, where its compression can lead to the formation of pre- or post-stenotic subclavian aneurysms and mural thrombus, often complicated by distal thromboembolisms that can affect the hands and/or fingers [7].

Duplex echography is the first-choice imaging technique used to detect vascular anomalies (aneurysms, stenosis, and thrombosis) due to its accessibility and cost [3]. A chest X-ray can also be used if bony abnormalities are suspected. Although these imaging techniques are used, computed tomography angiography (CTA) is considered the gold standard for diagnosis, as it can describe in detail the anatomy of the vessels. MRI can be indicated, particularly if the use of CTA is not advisable, and to exclude a nTOS component, which can be difficult to distinguish from aTOS or vTOS [1,4].

Treatment of aTOS requires resection of an atypical rib; two main surgical approaches are typically used: the transaxillary and supraclavicular [5,8]. The infraclavicular approach can also be used in association with the supraclavicular approach when arterial reconstruction is needed [4].

The purpose of this paper is to report a rare case of aTOS presenting as acute upper limb ischemia in a young patient with a cervical rib and additional thrombotic risk factors. Given the potential severity of its complications and the challenges in diagnosis, reporting such cases remains clinically relevant to improve recognition and optimize management strategies.

## Case presentation

In April 2024, a 19-year-old woman presented to her general practitioner with pain in the right hand that had started one week before, followed by coldness and numbness in the same hand, which had become worse more recently. She also experienced intermittent pallor and muscular pain during contractions in the neck area, specifically in the superior trapezius muscle.

Her anamnesis revealed the recent initiation of a combined estrogen-progestin oral contraceptive, which she had started just 15 days before. The general practitioner performed a Duplex ultrasound of her right upper limb, which revealed that the radial and ulnar pulses were absent and that the brachial pulse was strongly diminished.

She was subsequently admitted to the emergency room of our hospital to complete the diagnostic workup. After a blood test for complete blood cell count, hemostasis, D-dimers, and C-reactive protein, showing no abnormalities, a second Duplex ultrasound was performed that day, confirming the same findings as the previous examination. An arterial-phase CTA was then performed, highlighting a complete thrombosis of the proximal two-thirds of the right subclavian artery and of the right brachial artery at its origin.

Based on these findings, a diagnosis of upper limb ischemia was made and was treated by thrombectomy using a Fogarty catheter. The patient was hospitalized overnight in the ICU for monitoring, where antiplatelet and anticoagulant therapies were initiated.

The subsequent hematology consultation identified both the newly started contraceptive therapy and the repetitive arm movements she was required to perform as a fast-food employee as risk factors for this thrombotic event. Moreover, the report from the consultation advised ruling out any presence of a subclavian stenosis caused by a supernumerary rib. For this reason, a comprehensive workup was pursued, including imaging to detect bony abnormalities, a complete thrombophilia screening, which was negative, and a breast ultrasound, which confirmed the absence of any suspicious nodular lesions in the right breast.

**Figure 1:**
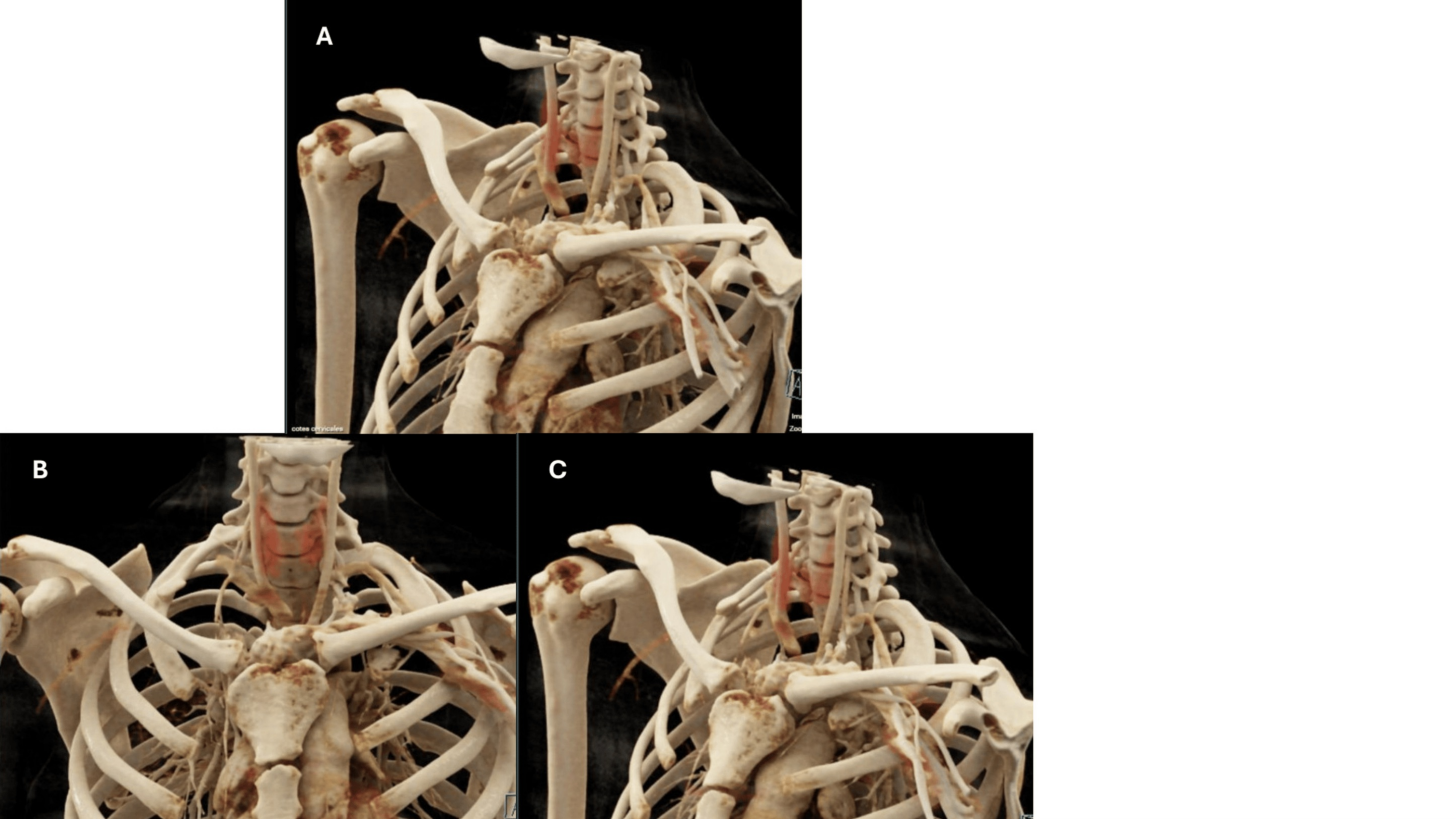
CT scan with three-dimensional reconstruction of the thoracic outlet before the cervical rib resection

A chest CT scan with three-dimensional reconstruction identified the presence of bilateral cervical ribs. The right cervical rib was complete, while the left one presented in an incomplete form (Figure 1). This finding established a link between the congenital anomaly and the acute thrombotic event, which initiated the involvement of thoracic surgery for the possibility of excising the right cervical rib.

The patient was then admitted to the thoracic surgery unit for the removal of the right cervical rib via a supraclavicular approach.

Surgical procedure

**Figure 2:**
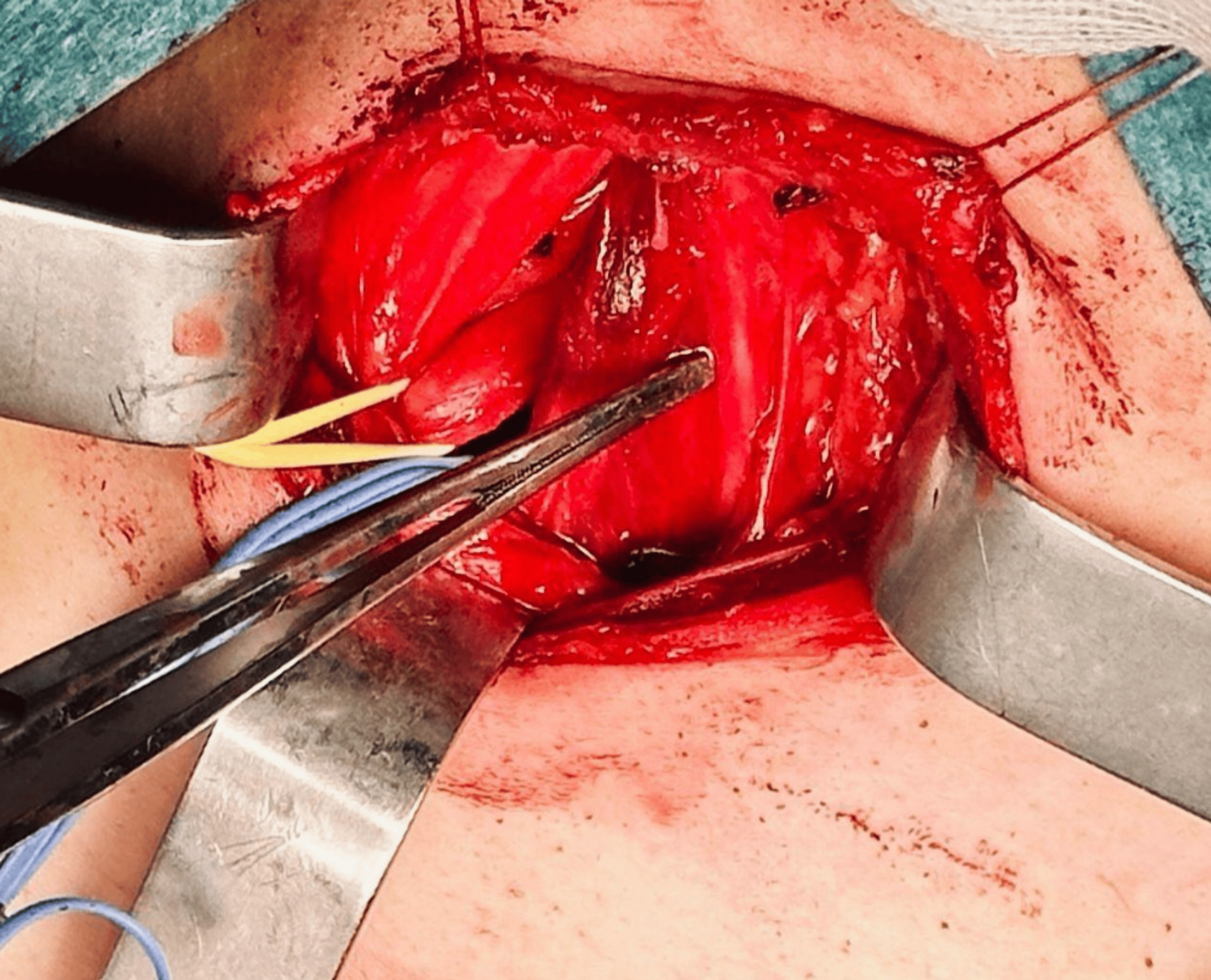
Supraclavicular approach

**Figure 3:**
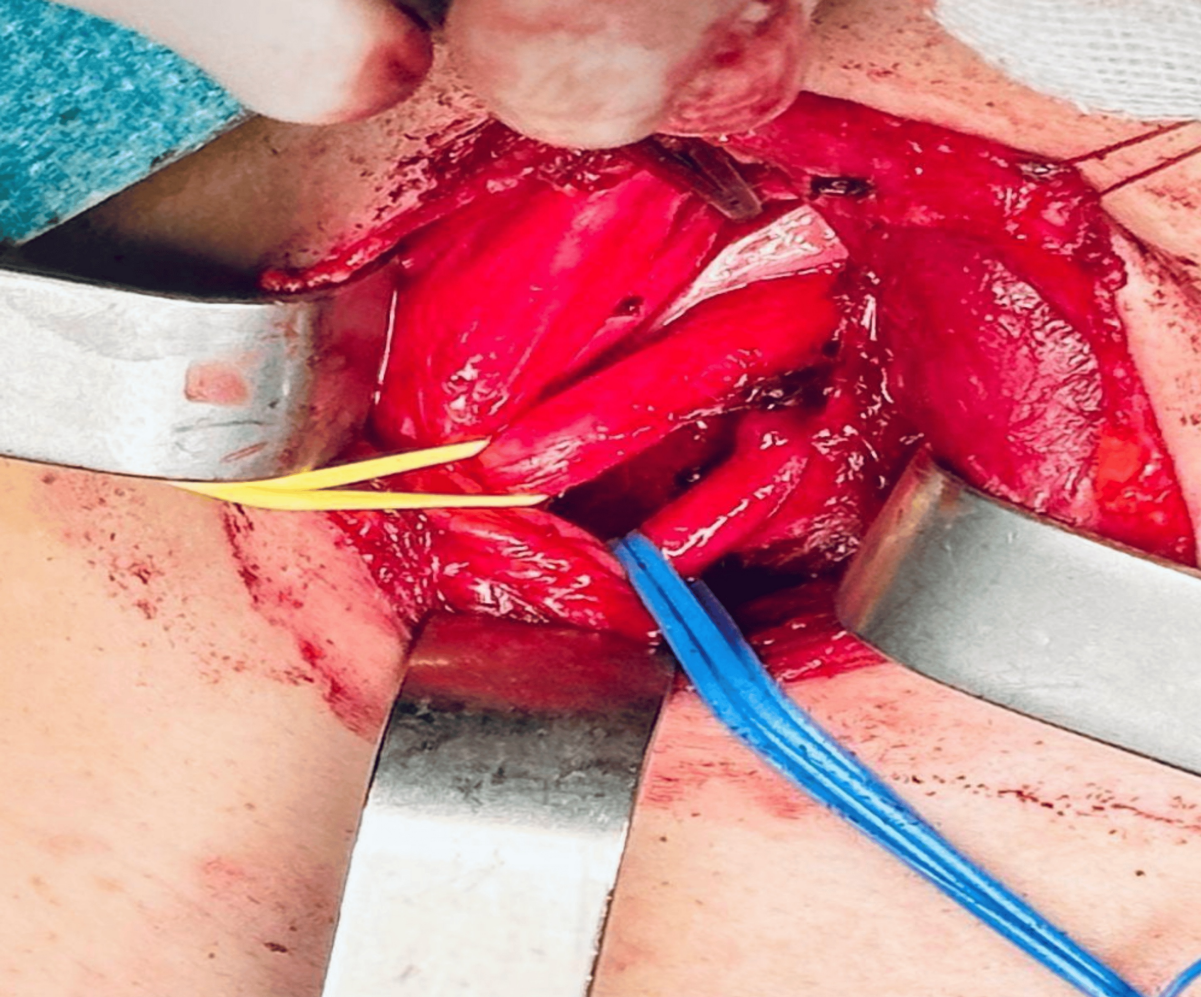
The blue vessel loop encircles the subclavian vein, while the yellow vessel loop encircles the subclavian artery.

**Figure 4:**
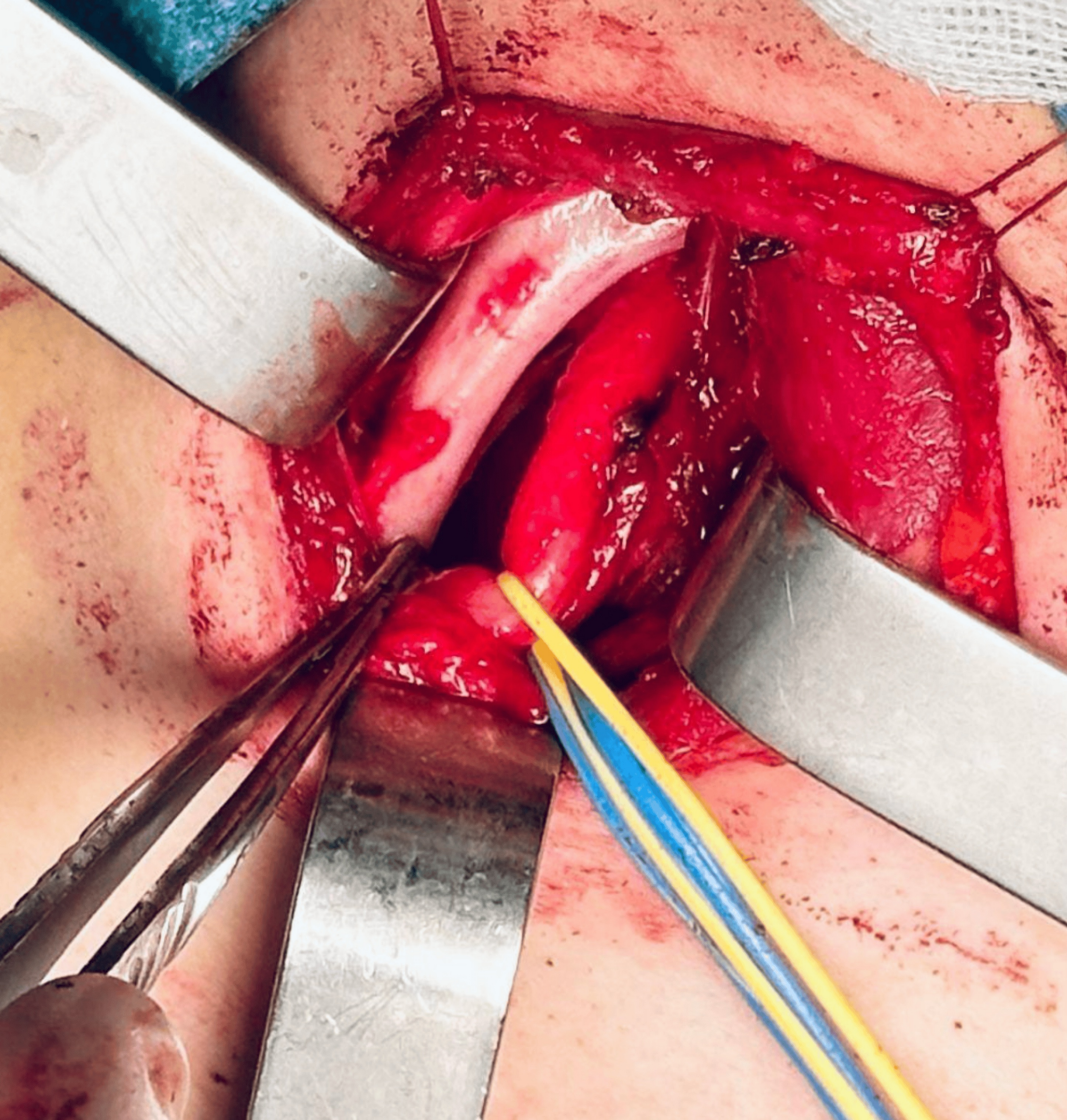
The surgical forceps indicate the supernumerary cervical rib.

The patient was positioned supine with the neck hyperextended and slightly rotated to the left. The surgical field was meticulously disinfected using chlorhexidine-alcohol, and standard sterile draping was applied. A supraclavicular incision was made over the supernumerary rib. The platysma muscle was incised, and the sternocleidomastoid muscle was retracted medially, providing access to the anterior scalene muscle and enabling identification of the phrenic nerve. The anterior scalene muscle was carefully retracted, exposing the brachial plexus branches, which were dissected and secured with silicone loops. The subclavian artery was also identified and looped for protection (Figures 2-4).

**Figure 5:**
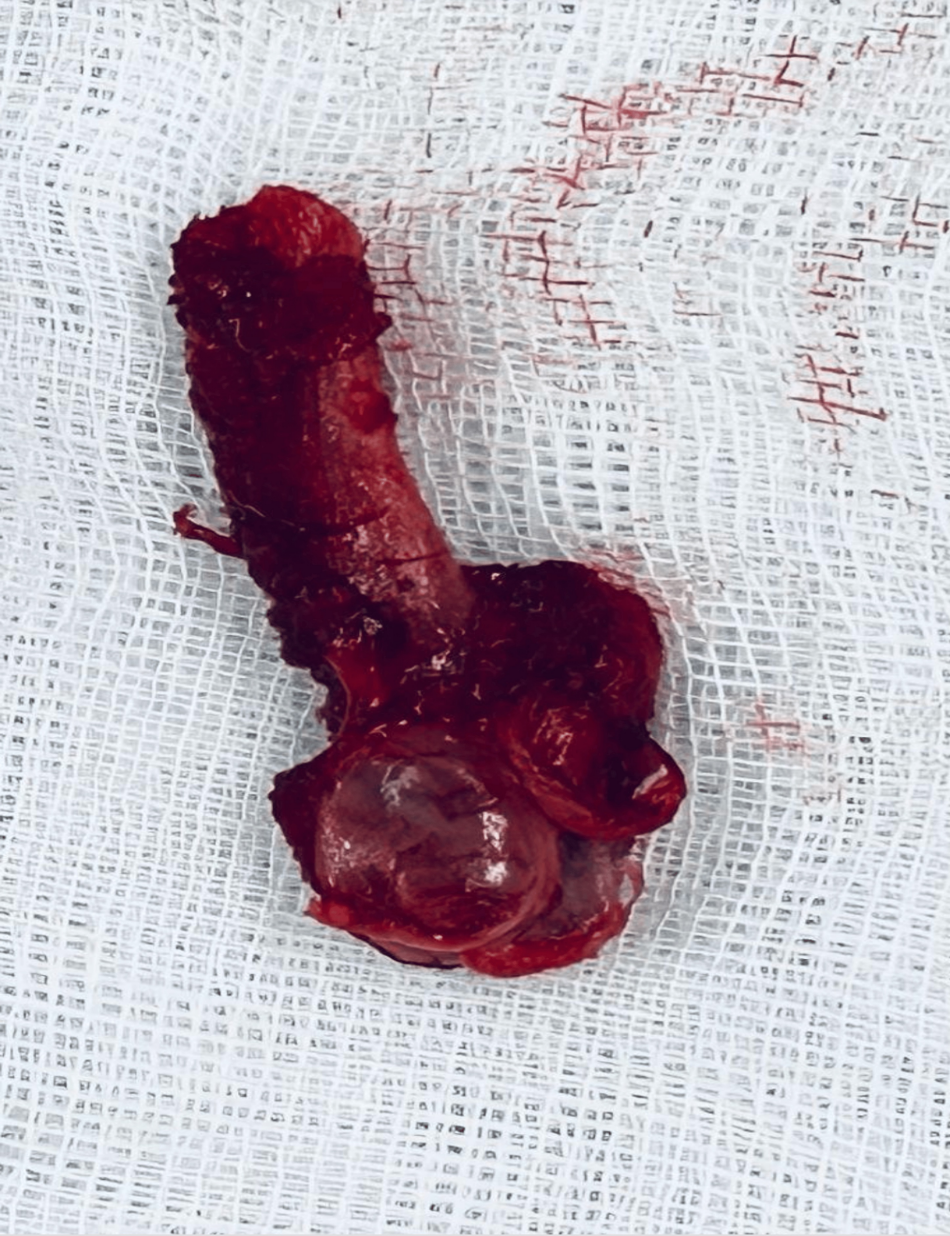
Resected cervical rib

With the neurovascular structures isolated, the supernumerary rib was progressively mobilized by detaching the insertions of the middle and anterior scalene muscles. The dissection continued posteriorly to the first rib. The rib was sectioned posteriorly at its articulation with the transverse process of C7 and disarticulated from its connection to the first rib. The resected rib was removed (Figure 5), and the surgical site was thoroughly inspected to ensure no residual bone fragments were in contact with the brachial plexus. Bone wax was applied to the resection site for hemostasis. Silicone loops securing the subclavian artery and the brachial plexus branches were removed, confirming the complete decompression of these structures. Hemostasis was verified, and the wound was closed in three layers: fascia, subcutaneous tissue, and skin.

## Discussion

TOS, including aTOS, can be particularly challenging to diagnose and formulate a treatment plan. Several factors contribute to this difficulty: aTOS is relatively uncommon and not widely recognized, it has a clinical presentation that can mimic other more common conditions, and it still lacks some standardized criteria to help physicians make the diagnosis with certainty [7]. Additionally, the prevalence of this syndrome is quite low, and this leads to limited epidemiological and clinical data.

The aim of this report is to present a clinical case of aTOS and to contribute to the existing literature on this condition by highlighting how this case aligns with or differs from current evidence regarding the diagnostic and therapeutic approach, from epidemiology to surgical management.

From a pathophysiological standpoint, aTOS results from chronic compression of the subclavian artery, leading to a cycle of inflammation, microhemorrhages, and fibrosis, a condition that can change the arterial wall, finally leading to stenosis, fibrotic artery, microembolic events, and thrombosis [9,10].

From an epidemiological standpoint, aTOS is the least common manifestation of TOS, presenting in only 1% of all TOS patients [2-4]. Regarding sex-based differences, while most of the studies state that there is no difference in prevalence based on sex [3,4], some recent reviews suggest that there might be a higher prevalence in females [11].

aTOS can have various causes, both congenital and acquired [4]. In our presented case, the main cause was congenital, specifically a complete right cervical rib, and a wider context of additional risk factors that may have contributed to the development of the thrombosis. Cervical ribs are supernumerary ribs that arise from the seventh cervical vertebra. While studies have stated that their prevalence in the general population is below 1%, additional data have shown that this value varies between 0.58% and 6.2%, depending on the population studied [12]. Historically, the first description and classification of cervical ribs were made in the 19th century by the Austrian anatomist Wenzel Gruber, and these were later adapted into a modern classification that defines four distinct types of cervical ribs based on imaging findings [13].

Among the various risk factors that can lead to aTOS, some are related to the lifestyle of the patients, such as sports requiring repeated extreme shoulder abduction or other repetitive arm movements, repeatedly narrowing the thoracic outlet [9]. This might have been the case for this patient, as she is employed in a fast-food restaurant, where one of her duties requires repetitive arm movements every day.

It is also important to highlight the fact that the patient had started a combined estrogen-progestin oral contraceptive therapy, an established thrombophilic regimen, just two weeks preceding the thrombotic event. There are no studies of aTOS that classify oral contraceptive drugs as a risk factor, but this points out how a complete patient history can help identify all the potential causes and risk factors for tailoring a therapeutic approach.

As previously mentioned, diagnosis of aTOS can be quite challenging. Typically, as in this case, this syndrome is rarely diagnosed in its earlier stages because the symptoms tend to be mild and easily overlooked, both by patients and physicians. Diagnostic delay can lead to more severe presentations, including major arterial occlusion and potentially limb-threatening ischemia [2,10]. The other clinical symptoms associated with aTOS are claudication, pain, upper limb or hand pallor, coldness, paresthesia, and digital ischemia [2].

In general, the physical examination of a patient with suspected aTOS requires a thorough assessment of the upper limb and cervical regions, including evaluation of the arm pulses at rest, the clinical presence of hand findings suggestive of arterial thromboembolic events, a neurologic examination, and the presence of signs such as ulcerations, gangrene, or pallor [4,14]. There are also some compression maneuvers, such as the Wright hyperabduction and the Adson’s test, that are traditionally used to provoke a decrease in arterial pressure in the upper limb, supporting the existence of vascular compression. However, their utility has been increasingly questioned because of their low specificity, resulting in many positive responses in asymptomatic patients creating a lack in diagnostic utility [3,4,15].

In this case, given the severity of the presentation, with a clinical scenario compatible with acute upper limb ischemia, the diagnostic process first focused on assessing the severity of the ischemia and the localization of the thromboembolic lesion, which was treated via catheterization. Only after intervention, the investigation shifted toward the research of the underlying cause of this event, identifying the supernumerary rib. It was also important to consider other possible conditions in the differential diagnosis, such as thrombophilia or breast cancer, that were excluded with an appropriate workup.

Imaging plays a key role in the diagnostic process of aTOS. Duplex ultrasound is commonly used when aTOS is suspected, since it is a fast, low-cost, and non-invasive examination that aids in the diagnosis by visualizing blood vessel alterations, such as aneurysms, stenosis, and thrombosis [3,4]. To identify the cause, radiography is particularly useful, as it can reveal the presence of supernumerary ribs, the main etiology of this syndrome, although it is often insufficient for a definitive diagnosis. In addition, chest CT can demonstrate potential narrowing of the thoracic outlet, and MRI can detect soft tissue abnormalities. However, angiographic techniques remain the gold standard for the diagnosis, both CTA and catheter-based arteriography [4,9].

In this case, a CT scan with 3D reconstruction led to the discovery of the complete cervical rib on the right and an incomplete one on the left side. This technique is an important tool both to identify the presence of cervical ribs, as it can be considered the gold standard for this type of bony abnormality [12], and also for their treatment, since it greatly helps surgeons better visualize the position of the rib and the severity of the thoracic outlet narrowing, allowing for a more optimal surgical treatment.

Management and treatment of this syndrome are guided by the Sher classification [4]. Regarding surgical treatment, there are three main approaches for cervical rib resection: transaxillary, supraclavicular, and infraclavicular [4,5,8].

In the presented case, the supraclavicular approach was selected. Although the choice of the surgical approach is typically based on the surgeon’s preference, the supraclavicular approach tends to be favored since it can provide better exposure of both bony abnormalities and the subclavian artery [10].

After the surgical treatment, the patient must be positioned in a 30-degree head-up position, and vital signs must be monitored [16]. A chest X-ray should be performed to assess the quality of the rib excision, detect any pneumothorax or hemothorax, and evaluate the position of the diaphragm, which can reveal the presence of phrenic nerve injury. Chest X-rays should be performed both in the recovery room and on the first morning after surgery [8]. If a drain is placed during the surgery, it is typically removed within 24 to 48 hours. It is also important to initiate range-of-motion exercises, preferably supervised by a physiotherapist, starting from the first postoperative day during the hospitalization, which normally lasts one to two days [8,16].

**Figure 6:**
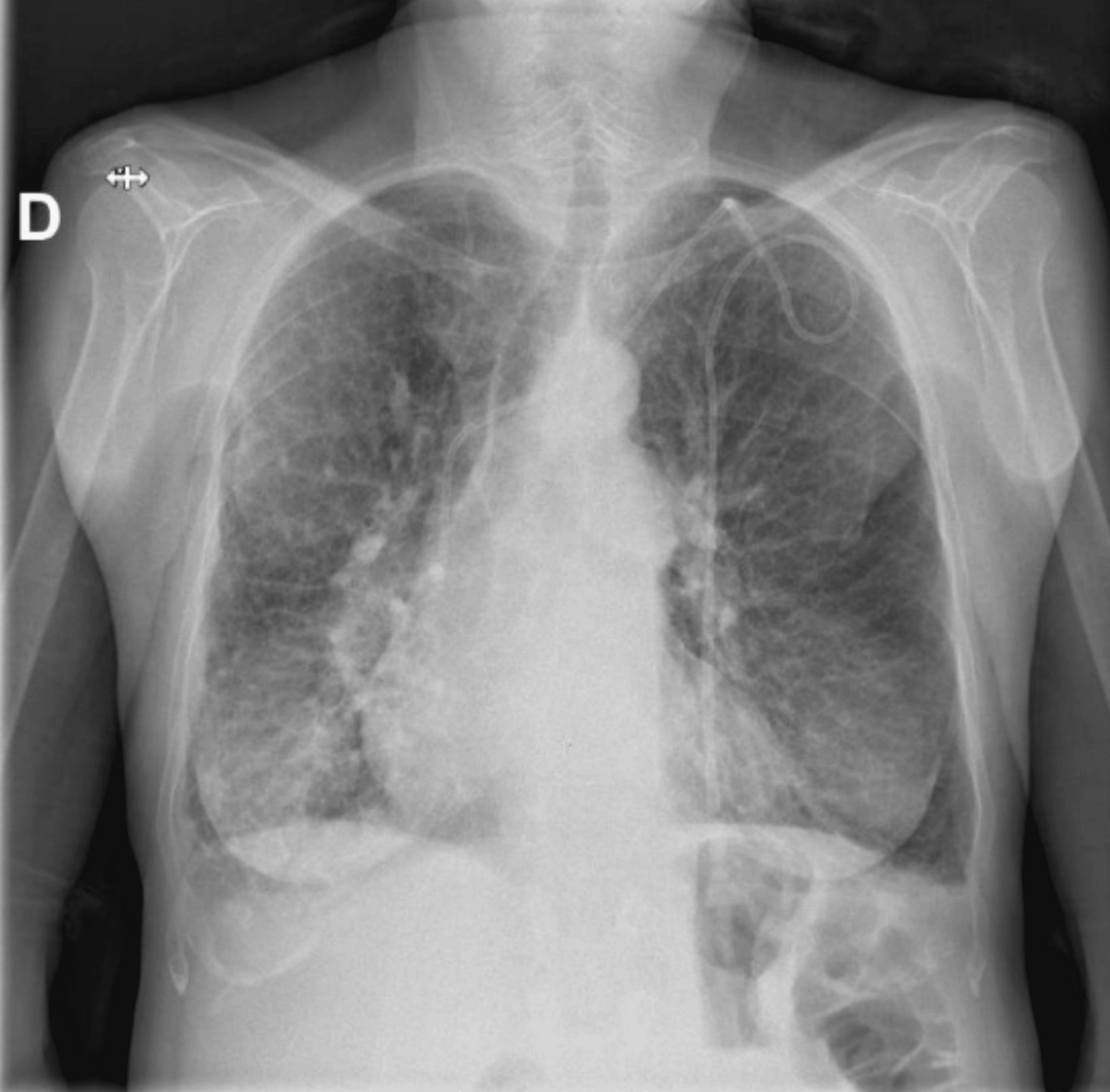
Post-operatory radiography

In this case, the patient responded well to the surgery. The chest X-ray showed the complete resection of the right cervical rib and the absence of other surgical complications (Figure 6). After starting physiotherapy in the hospital, she was discharged with instructions to continue physiotherapy at home.

## Conclusions

In conclusion, this case highlights how aTOS is still a subtle condition that is often diagnosed when the symptoms become very severe, and it also demonstrates how underlying causes can affect even young patients, especially in a context of multiple risk factors. The diagnostic assessment of this syndrome requires a multidisciplinary team to correctly establish the link between the syndrome and the underlying cause. Concerning the treatment of cervical ribs, the supraclavicular approach continues to be the preferred and most efficient method, resulting in effective decompression of the thoracic outlet and a reduced risk of recurrence.
